# Contribution of XN‐10 Sysmex Parameters in the Cytological Monitoring of Acute Promyelocytic Leukemia

**DOI:** 10.1111/ijlh.70031

**Published:** 2025-11-24

**Authors:** Lucille De Maria, Logan Baldini, Joy Mouanes Abelin, Corinne Ferrero, Yann Boursier, Yael Berda‐Haddad, Pierre Toulon

**Affiliations:** ^1^ Centre Hospitalier Universitaire de Nice Nice France; ^2^ Centre Antoine Lacassagne Nice Nice France; ^3^ Assistance publique des hôpitaux de Marseille Marseille France

**Keywords:** acute promyelocytic leukemia, cytological monitoring, Sysmex haematology equipment

## Abstract

**Objectives:**

The all‐trans‐retinoic acid (ATRA) treatment used in acute promyelocytic leukemia (APL) has the particularity of inducing differentiation of the leukemia cells. As a result, within the same smear, several stages of cellular differentiation of the granular lineage are present, which can make it difficult to distinguish elements when counting cells.

**Methods:**

The aim of this study is to use the technical data from Sysmex haematology equipment in patients with APL to improve their cytological monitoring and harmonize blood count results from one operator to another. We collected 132 samples from patients with APL over a period of 45 days from the day of diagnosis and divided them into two groups: presence or absence of blast cells on the blood smear.

**Results:**

Seven parameters of leukocyte subpopulations were selected for cytological monitoring. We developed a decision algorithm based on these, including decision thresholds for predicting the presence or absence of blasts. These data were verified on a validation cohort of 110 samples with APL, set up at a different site from the training cohort. The algorithm predicted the presence of blast cells in these samples with a sensitivity of 84.6% and a specificity of 86.7%.

**Conclusion:**

In the context of cytological monitoring of patients with APL, the study of technical data has not been developed yet, despite the fact that this is a difficult and sometimes nonhomogeneous task from one operator to another. The proposed algorithm could be a facilitating tool in cytology to harmonize practices both within and between sites.

## 1 Introduction

Acute promyelocytic leukemia (APL), also known as M3 acute myeloid leukemia (AML) in the French American British (FAB) classification, accounts for 10%–15% of adult AML and 5%–10% of childhood AML. Incidence is roughly the same in both sexes, with a median age at diagnosis of 57 in men and 54 in women [1].

Therapeutic management is an emergency due to the high risk of hemorrhage. It is based on the use of all‐trans‐retinoic acid (ATRA), which allows leukemic promyelocytes to differentiate into mature granulocytes [2, 3].

This implies that within a single blood smear, there are several stages of cellular differentiation of the granulocyte lineage. This makes it difficult to count cell populations and standardize them between different operators in the same laboratory.

The aim of this work is to improve cytological monitoring of these patients, using positional technical parameters for the various leukocyte subpopulations, in particular with Sysmex XN10 analyzers.

By combining certain parameters supplied by the automated system, these data help the cytologist to harmonize the rendering of immature granulocytes and residual blastosis in order to achieve maximum reproducibility between different operators in the same laboratory [4, 5].

## 2 Material and Methods

Patient samples were collected within 45 days of diagnosis; all were confirmed in cytogenetics and molecular biology. Treatment with ATRA was initiated as soon as possible (< 72 h), following the diagnosis of APL on blood cytology.

In this work, we set up two cohorts: one training cohort, which enabled us to draw our initial conclusions, and one validation cohort, which enabled us to verify these results.

A complete blood count (CBC) was performed at diagnosis in all patients in the cohort, including erythrocyte, platelet and leukocyte counts.

The training cohort includes 132 samples from five adult patients and one child whose diagnosis and follow‐up of APL was performed in Marseille, at the Timone Hospital between February 2023 and June 2024. Of six patients, four were leukopenic at diagnosis.

The median age was 42 years (range 22–89 years), with the age of the child in the cohort excluded (6 years) and the group included three females and three males.

There was no significant difference (*p* > 0.05, Mann–Whitney test) for the technical parameters of Sysmex equipment studied between the values of the adult and child samples in the cohort.

The validation cohort includes 110 samples from six adult patients diagnosed with APL at the Nice University Hospital between January 2023 and September 2024.

Among these patients, five were leukopenic and 1 hyperleukocytic.

The median age was 71.5 years (range 44–86 years), and the group included three females and three males.

Samples were taken from whole blood on a 4 mL K3 Ethylene Diamine Tetra Acetic (EDTA) tube. They were analyzed on the XN‐10 Sysmex (Kobe, Japan), which is qualified according to the laboratory's good practices (calibration, daily quality control).

These analyzers use flow cytometry after specific labeling of nucleic acids with a fluorochrome to establish a leukocyte formula including neutrophils (NE), eosinophils (EO), basophils (EB), lymphocytes (LY), monocytes (MO) and immature granulars (IG).

Each cell is identified according to three technical parameters supplied by the WDF channel of the equipment: its structure (*X*‐axis or SSC), its fluorescence (*Y*‐axis or SFL) and its size (*Z*‐axis or FSC).

For each sample, the analyzer provides the median position on the three axes of NE (NE‐X, NE‐Y, NE‐Z), LY (LY‐X, LY‐Y, LY‐Z) and MO (MO‐X, MO‐Y, MO‐Z), as well as their dispersion (NE‐WX, NE‐WY, NE‐WZ/LY‐WX, LY‐WY, LY‐WZ/MO‐WX, MO‐WY, MO‐Z). The dispersion attributed to each population is the ratio of the width of the cell cloud to the value of its median position on the SSC axis.

Details of the leukocyte sub‐populations and all the technical positioning parameters used to classify them in the various scattergrams are listed.

A blood smear was taken according to the laboratory's rules of expertise, in particular in the event of quantitative and/or qualitative anomalies, and stained with May‐Grünwald Giemsa (MGG) using an automatic stainer (SP‐50 XN Sysmex).

Blood smears were analyzed under the optical microscope by different laboratory operators.

There was no significant age difference between the two cohorts, Mann–Whitney test (*p* = 0.092), *p* > 0.05.

As the values studied do not follow a normal distribution (Shapiro–Wilk test), a Mann–Whitney test was used to compare the two groups (presence of blasts/absence of blasts) for each of the parameters studied. A *p*‐value of less than 0.05 was considered statistically significant.

Boxplots and ROC (receiver operating characteristic) curves were plotted using GraphPad Prism8 software to determine the parameters that could be used and their associated threshold values to discriminate a blastic sample from a non‐blast sample.

Algorithm performance was assessed using a leave‐one‐patient‐out cross‐validation approach, where all measurements from one patient were excluded from model training and used for validation.

This procedure was applied both with predefined cut‐offs and with data‐driven cut‐offs determined from the training set using the Youden index derived from ROC curve analysis.

Model robustness was further evaluated by bootstrap resampling (1000 iterations, patient‐level sampling with replacement). Sensitivity and specificity were computed for each iteration, and 95% confidence intervals were derived from the empirical bootstrap distributions.

## 3 Results

A total of 132 samples from the 6 patients treated with ATRA in the training cohort were analyzed, with all technical parameters and analytical alarms recovered.

They were divided into two groups according to the presence of blasts: 73 blastic leukocyte formulas and 59 non‐blastic leukocyte formulas as shown in Table 1.

**TABLE 1:** Number of samples per patient in the training cohort. This table shows the number of samples taken per patient and the distribution of samples containing blasts or not combined with myelimia or not.

	Number of samples	Number of formulas with blasts cells		Number of formulas without blasts cells
		Blast cells alone	Immature granulocytes and blasts	
Patient 1	22	3	6	13
Patient 2	16	4	9	3
Patient 3	26	8	5	13
Patient 4	23	12	5	6
Patient 5	22	6	4	12
Patient 6	23	0	11	12
Total	132	73		59

Based on current literature [6, 7] and mathematical models using leukocyte subpopulation positioning/dispersion parameters, 12 parameters were statistically tested to determine which would be most relevant for cytological monitoring of the APL cohort. These were:

– *for the granular*: positioning parameters NE‐SFL (NE‐Y), NE‐FSC (NE‐Z), NE‐SSC (NE‐X) as well as the quantitative parameter “IG‐XN” corresponding to the immature granular population above the NE cloud, the “Left‐Shift” corresponding to the detection of unsegmented granulocytes (“band cells”) and NE‐GI alarms corresponding to NE activation status.
– *for lymphocytes*: LY‐Z positioning and LY‐WY dispersion parameters
– *for monocytes*: positioning parameter MO‐X and dispersion parameters MO‐WX, MO‐WY, MO‐WZ.

Statistical study of the cohort shows that for seven of the initial 12 parameters, a significant difference (Mann Whitney, *p* < 0.05) exists between the two subgroups (blastic and non‐blastic samples).

These parameters are IG‐XN, Left‐Shift, NE‐SFL, NE‐FSC, MO‐WX, MO‐WY and MO‐WZ. The corresponding ROC curves are shown in Appendix A.

For each of the 132 samples, raw data for the seven discriminant parameters were collected, yielding seven quantitative variables per sample, linked to a single qualitative variable: presence or absence of blasts.

Two quantitative thresholds for each parameter were defined to obtain the best possible correlation with the presence/absence of blasts for a given sample.

– The first is a very high value, beyond which the sensitivity of blasts is 100%. This means, if one of the seven parameters exceeds its “unconditional” threshold, the presence of blasts can be confirmed. By reducing the number of false negatives, they increase the sensitivity and negative predictive value of our decision algorithm (see Appendix B).
– The second, called the “defined threshold,” corresponds to a lower value than the unconditional threshold, and requires the combination of at least four of the seven parameters to confirm the persistence of blasts on cytology. This minimum of four of the seven parameters corresponds to the best compromise of sensitivity and specificity for the detection of blast cells.

These threshold values for each parameter were chosen using ROC curves, and correspond to the best compromise of sensitivity and specificity for each threshold of each parameter. The results are summarized in Table 2.

**TABLE 2:** Summary table of the threshold definitions. The following summary table shows the threshold definitions applied to IG‐XN, NE‐SFL, left shift, MO‐WX, MO‐WY, MO‐WZ and NE‐FSC: The “unconditional” threshold, where a single parameter exceedance indicates blasts, and the “defined” threshold, where at least four parameter exceedances suggest persistent blastosis.

	Unconditional threshold	Defined threshold
IG‐XN	> 23	> 2.2
NE‐SFL	> 65	> 41.45
Left shift	> 230	> 15.0
MO‐WX	> 538	> 289.5
MO‐WY	> 1057	> 787.5
MO‐WZ	> 805	> 705
NE‐FSC	< 67	< 72.6

Note that using fewer than seven parameters to detect the presence of blasts makes the results much less reliable.

**FIGURE 1:**
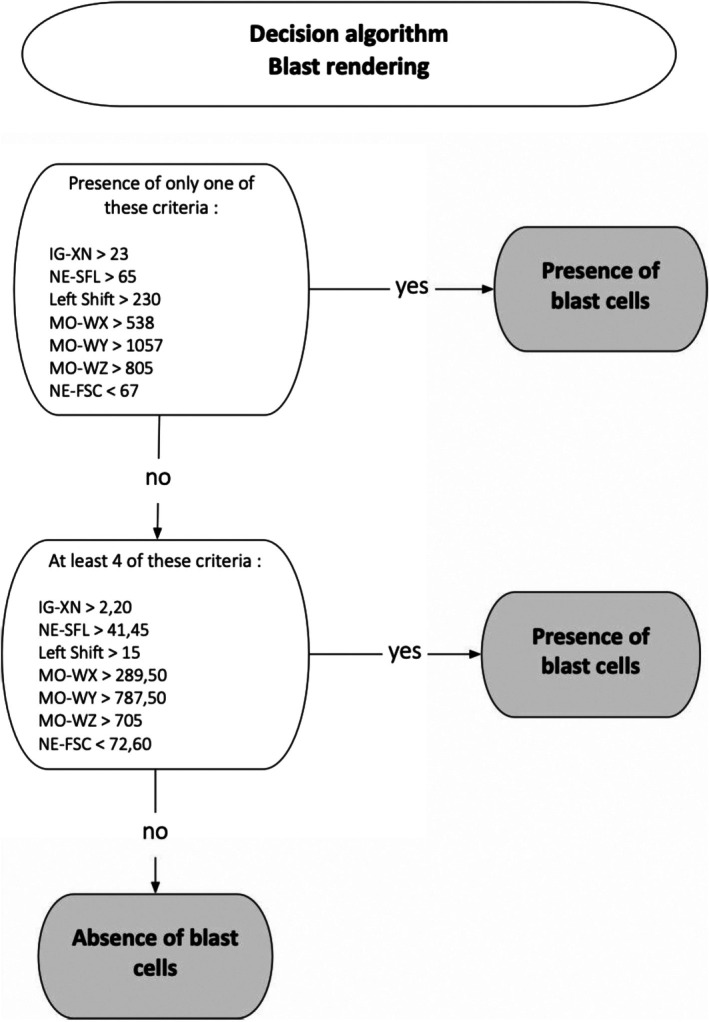
Decision algorithm for blood blast status according to the defined parameters of interest.

These observations led to the development of a decision tree presented in Figure 1.

It predicts the presence of blasts in a sample from a patient followed up for APL, with a sensitivity of 89%, a specificity of 86%, a PPV of 86% and an NPV of 89% in the training cohort.

For samples with low blastosis between 1% and 10%, representing 17 samples, the algorithm performed equally well, with a sensitivity of 76%.

There was no significant difference between the overall sensitivity taking into account all samples and the sensitivity of samples with blasts < 10% (*p*‐value = 0.134, comparison of proportions).

The bootstrap‐corrected performances were consistent with those obtained on the original training cohort, with a sensitivity of 0.887 (95% CI: 0.81–0.96) and a specificity of 0.866 (95% CI: 0.77–1.00), compared with a sensitivity of 0.89 and a specificity of 0.86 on the training data. These results indicate excellent model stability, suggesting no significant overfitting and supporting the generalizability of the decision algorithm.

This algorithm was tested on a validation cohort of 6 patients; cytologically, two of them exhibited a variant form of APL with sparse granulation. This represents 110 samples analyzed: 65 blastic leukocyte formulas and 45 non‐blastic leukocyte formulas with recovery of the technical parameters and analytical alarms contained in the algorithm. The algorithm has not yet been automated by artificial intelligence and was applied manually. Details of the number of WBC formulas per patient are given in Table 3.

**TABLE 3:** Number of blood cell counts per patient in the validation cohort according to peripheric blastosis.

	Number of sample	Number of formulas with blasts cells	Number of formulas without blasts cells
Patient 7	17	6	11
Patient 8	5	5	0
Patient 9	23	14	9
Patient 10	21	13	8
Patient 11	29	16	13
Patient 12	15	11	4
Total	110	65	45

Application of the algorithm enabled us to predict the presence of blasts in these samples with a sensitivity of 84.6% and a specificity of 86.7%. All false‐negative samples in this cohort came from two of the six patients, demonstrating very good sensitivity for the other 4.

A comparison of proportions test was performed; there were no significant differences between the two cohorts (*p*‐values > 0.05): the algorithm performed as well in the training cohort as in the validation cohort.

## 4 Discussion

This work has been inspired by the literature on the usefulness of hematology analyzer parameters in rapidly discriminating APL from other types of AML, as reported by Mishra et al. [6] and Park et al. [7].

In this work, we were interested in finding the combination of technical parameters that could help us in the rendering of the leukocyte formula in APL patients treated with ATRA+/− ATO.

Indeed, cytological monitoring is difficult, because unlike other chemotherapies used in acute leukemia, ATRA does not lyse promyelocytic blast cells but induces their differentiation. Blast cell decrease can take up to several weeks. Throughout this period, in the hospital environment, blasts and the immature granulocytes resulting from their differentiation co‐exist on the smear, making counting difficult.

For the seven parameters selected, two threshold values were defined: a so‐called unconditional threshold which, if exceeded for a single parameter, results in the presence of blasts on the smear and a lowest threshold which requires the combination of four parameters exceeding this threshold to point to the persistence of blood blastosis. The algorithm we've developed, using these threshold values, makes it possible to predict the presence or absence of blasts on the blood smear in the event of cytological doubt. It has the advantage of being equally effective in cases of low blastosis (1%–10%). However, it is not applicable to leukemias other than APL.

One of the limitations of our defined thresholds lies in the fact that the formulas were rendered by six different operators, and a re‐analysis of the slide by the same operator was necessary for some samples.

We investigated whether the technical parameters could also be used to determine the presence of immature granulocytes. It turned out that no parameter showed any significant change in the presence or absence of immature granulocytes, whether combined with the presence of blasts or not.

This can be explained by the fact that in the first few days of monitoring, the clouds of cell populations are sometimes poorly delineated and can overlap, giving rise to results that are sometimes aberrant and not usable.

To verify the algorithm's performance, we applied it to a validation cohort of 6 patients with APL. This cohort was set up at a different site from the training cohort, with new operators to limit the bias associated with cytological harmonization that can occur within the same site.

The results obtained are promising, with the algorithm's performance showing a sensitivity of 84.6% and a specificity of 86.7%.

We now need to continue using this algorithm in order to confirm its performance and feasibility in the daily practice of hospital laboratories.

## 5 Conclusion

Nowadays, we have an increasing amount of data that can be used in cases of doubt in blood cytology.

Cytology specialists are rare, and there are more and more developments in smear interpretation aids.

The use of technical data from hematology analyzers is on the increase, regardless of the automaton supplier, to assist in the reading of blood smears.

In the context of cytological monitoring of patients with APL, the study of technical data has not been developed yet, despite the fact that this is a difficult and sometimes non‐homogeneous task from one operator to another.

Thus, the proposed algorithm could be a facilitating tool in cytology to harmonize practices both within and between sites.

## Author Contributions

Lucille De Maria designed the study, analyzed data, and wrote the manuscript. Logan Baldini analyzed data and wrote the manuscript. Joy Mouanes Abelin, Corine Ferrero and Pierre Toulon wrote parts of the manuscript. Yann Boursier analyzed data. Yael Berda‐Haddad designed the study and wrote parts of the manuscript.

## Funding

The authors have nothing to report.

## Ethics Statement

The authors have nothing to report.

## Consent

The authors have nothing to report.

## Conflicts of Interest

The authors declare no conflicts of interest.

## Figures and Tables

**FIGURE A1:**
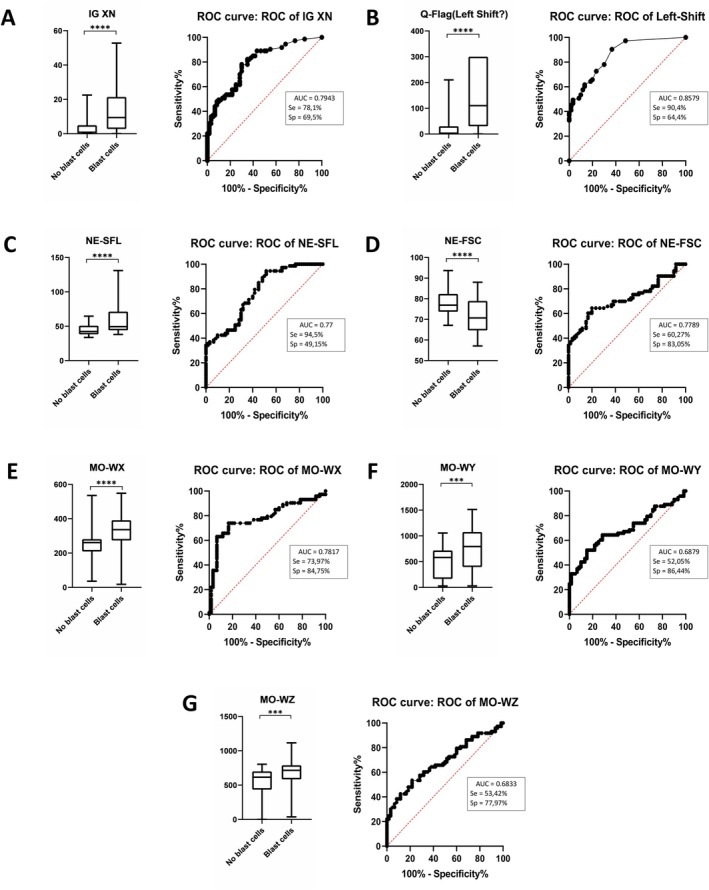
Blast cells cohort *n* = 73/no blast cells cohort *n* = 59. The ROC curves are a graphical representation of the thresholds chosen, corresponding to the best compromise between sensitivity and specificity for each parameter with regard to the presence or absence of blasts. (A) IG‐XN, *p* value < 0.0001, Mann–Whitney test. (B) Leftshift, *p* value < 0.0001, Mann–Whitney test. (C) NE‐SFL, *p* value < 0.0001, Mann–Whitney test. (D) NE‐FSC, *p* value < 0.0001, Mann–Whitney test. (E) MO‐WX, *p* value < 0.0001, Mann–Whitney test. (F) MO‐WY, *p* value 0.0002, Mann–Whitney test. (G) MO‐WZ, *p* value 0.0003, Mann–Whitney test. ns, *p* > 0.05 (non significative), *p ≤ 0.05, **p ≤ 0.01, ***p ≤ 0.001, ****p ≤ 0.0001.

## Data Availability

The data that support the findings of this study are available from the corresponding author upon reasonable request.

## Appendix A Statistical Evaluation of Selected Parameters of Interest

See Figure A1.

## Appendix B Benefits of Using Unconditional Thresholds to Improve the Decision Algorithm in the Training Cohort

**Table:**

	TP	TN	FP	FN	Se	Sp	PPV	NPV
4 concordant parameters	60	51	8	13	82%	86%	88%	79%
With unconditional thresholds	65	51	8	8	89%	86%	89%	86%
